# Editorial: Dynamic Emotional Communication

**DOI:** 10.3389/fpsyg.2019.02836

**Published:** 2019-12-17

**Authors:** Wataru Sato, Eva G. Krumhuber, Tjeerd Jellema, Justin H. G. Williams

**Affiliations:** ^1^Kokoro Research Center, Kyoto University, Kyoto, Japan; ^2^Department of Experimental Psychology, University College London, London, United Kingdom; ^3^Department of Psychology, University of Hull, Hull, United Kingdom; ^4^Translational Neuroscience Group, Institute of Medical Sciences, University of Aberdeen, Aberdeen, United Kingdom

**Keywords:** action observation network, body action, dyadic interaction, dynamic facial expression, emotion recognition

## Introduction

Psychological research has a long history of investigating facial and bodily expressions associated with emotion. This is partly due to the fact that non-verbal behaviors are indispensable communicative signals during the creation and maintenance of social relationships. A number of neuroscientific studies have also investigated the neural mechanisms underlying the processing of these emotional signals.

However, most previous research assessing emotional communication has been conducted using static stimuli. Although researchers have accumulated valuable information about the psychological and neural mechanisms underlying the processing of emotional signals using such stimuli, their static nature may have left important phenomena unexamined.

To address this issue, recent studies have explored emotional communication using dynamic facial and bodily expressions of emotion, which has had important consequences for emotion research. Because dynamic emotional expressions are associated with increased ecological validity, resulting in a number of important differences in the psychological/neural processing between dynamic and static information, a host of novel aspects of emotional communication have been elucidated. Furthermore, the dynamic perspective can be applied to broader methodological and conceptual areas.

The present Research Topic brings together a collection of new articles that have investigated dynamic emotional communication and demonstrates recent advances in this field of research. Here, we introduce these articles and discuss them in the context of related studies by grouping them into the following four areas: (a) decoding of dynamic emotional signals, (b) moderators of dynamic emotional signal decoding, (c) encoding of dynamic emotional signals, and (d) other dynamic aspects of emotional communication. The term “decoding” was used to refer to various types of processing (e.g., perceptual and motor) in addition to the recognition of emotions. The term “encoding” was used to refer to the production of emotional signals.

## Decoding of Dynamic Emotional Signals

Seminal research has demonstrated that emotional recognition based on dynamic facial expressions is more efficient than that based on static expressions (Bassili, 1978), with several subsequent studies investigating this issue (for a review, see Krumhuber et al., 2013; Krumhuber and Skora, 2016). In this Research Topic, Dobs et al. reviewed the literature and reported that there are evident dynamic advantages for subtle expressions or for full-blown expressions under suboptimal conditions. Additionally, these authors provided an overview of the methods used to present dynamic facial expressions (e.g., videos and point lights) as well as their advantages and disadvantages.

Several studies have reported that the genuineness of an emotional message is decoded more effectively from dynamic, compared with static, facial expressions. For example, Zloteanu et al. investigated the discrimination performance of genuine expressions vs. deliberate expressions of surprise that were presented in both dynamic and static formats. These authors found that dynamic genuine expressions are perceived as more genuine-looking than static ones and that the presentation format modulated the genuineness ratings of deliberate expressions. In a similar vein, Namba et al. investigated whether decoders could distinguish between genuine and deliberate facial expressions of some emotions when they are presented in dynamic and static formats. The discriminability of the genuineness of an expression was enhanced for dynamic displays, in comparison to static displays. Busin et al. assessed the judgements of genuine vs. masked emotions in dynamic facial expressions rotated to the left or right side. Eye movement patterns revealed preferential attention to the left hemi-face, which has been previously reported during the processing of static expressions. Other studies have revealed that the dynamic nature (e.g., speed) of facial expressions provides information about the naturalness (Sato and Yoshikawa, 2004), genuineness (Krumhuber and Kappas, 2005), and trustworthiness (Krumhuber et al., 2007) of the portrayed emotion.

Various types of other information can be decoded from dynamic emotional signals. Orlowska et al. evaluated the recognition of reward, affiliative, and dominance smiles during dynamic and static presentations and found that the recognition of affiliative smiles is more accurate for dynamic expressions than static expressions. The authors also assessed the effects of facial muscle restriction and suggested that facial mimicry is unlikely to be critical to this process. Other studies have shown that, compared with static expressions, dynamic facial expressions facilitate the detection of an expression (Ceccarini and Caudeka, 2013), the experience of emotional arousal (Sato and Yoshikawa, 2007a), and facial mimicry (Weyers et al., 2006; Sato and Yoshikawa, 2007b). Different visual styles between dynamic and static facial expressions have been suggested in the context of eye fixation patterns (e.g., more fixation on the center for dynamic expressions; Blais et al., 2017).

Some studies have investigated multimodal dynamic emotional signals, which are more natural than those from a single modality. Garrido-Vásquez et al. recorded event-recorded potentials (ERPs) to investigate the priming effects of dynamic facial expressions (angry, happy, and neutral) on the processing of emotionally intoned sentences (angry and happy). The amplitudes of auditory-related components at ~100 ms are higher in response to incongruently primed sentences than other conditions, suggesting the occurrence of rapid cross-modal emotional interactions. Mortillaro and Dukes reviewed studies investigating the decoding and encoding of facial and bodily expressions of positive emotions. They proposed that the inclusion of dynamic information and facial as well as bodily signals is important when distinguishing between expressions of positive emotions (e.g., joy and pride).

Valid stimulus sets are needed to investigate the decoding of emotional signals. For this purpose, Calvo et al. developed a database of dynamic emotional facial expressions by creating morphing animations. They validated these novel stimuli via human observer judgments as well as automated assessment of facial expressions. Several other studies have developed stimulus databases (for a review, see Krumhuber et al., 2016), allowing for the selection of an appropriate database based on the researcher's needs.

A number of neuroimaging studies have investigated the neural mechanisms underlying the processing of dynamic emotional signals (e.g., Sato et al., 2004). Zinchenko et al. conducted meta-analysis of functional magnetic resonance imaging (fMRI) studies including dynamic facial expressions. They found that some brain regions (e.g., the fusiform and middle temporal gyri, amygdala, and inferior frontal gyrus) are robustly activated during the observation of dynamic facial expressions. The involvement of action observation network (AON; e.g., the middle temporal gyrus/superior temporal sulcus and inferior frontal gyrus), which can match the observation and execution of actions (cf. Rizzolatti et al., 2001), appears to be one of the most distinctive features associated with the neural processing of dynamic, compared with static, facial expressions. To further investigate this issue, Rymarczyk et al. simultaneously recorded facial electromyography (EMG) and fMRI data during the observation of dynamic and static facial expressions of fear and disgust. They reported that facial EMG patterns of facial mimicry are correlated with specific activation in several brain regions, including the AON, under dynamic presentation conditions. There are several other unique aspects of the neural processing of dynamic facial expressions compared with that of static expressions. For example, the observation of dynamic facial expressions evidently induces modulatory influences from the amygdala to the neocortex (Sato et al., 2017) and clearly reveals hemispheric functional asymmetry (right cortical and left cerebellar; Sato et al., 2019). Differences in the decoding of dynamic and static facial expressions have also been suggested by lesion studies (e.g., Humphreys et al., 1993).

Several neurophysiological studies in animals have provided information about the cellular-level neural substrates involved in dynamic emotional signal decoding. For example, Jellema and Perrett (2003) found that some neurons in the superior temporal sulcus of monkeys fire in response to dynamic bodily actions but not to static postures.

## Moderators of Dynamic Emotional Signal Decoding

Several stimulus properties of dynamic emotional signals moderate the decoding processes. For example, Plouffe-Demers et al. compared spatial frequency tuning during the recognition of dynamic and static facial expressions. The results showed that the recognition of dynamic facial expressions relies more strongly on lower spatial frequencies. Rooney and Bálint tested the effects of shot scale (i.e., the apparent distance of characters from the camera) on the tendency to recognize the mental states of others in fictional films. Close-up, compared with long, shots of a character are associated with higher tendencies to attribute emotional and mental states to a character.

Perceiver factors also moderate the decoding process of dynamic emotional signals. Wingenbach et al. investigated the effects of manipulating facial muscles on the recognition of emotion from dynamic facial expressions. Compared to passive viewing, holding a pen in the mouth reduces recognition accuracy of facial expressions based on salient features in the lower face region (e.g., happy expressions), indicating that bodily actions shape the processing of dynamic facial expressions. In a similar vein, Kato et al. explored the role of manual movements in the perception of valence from emotional scenes. Downward manual movements (temporally proximate and after the observation of images) made the scenes appear more emotional negative. Other studies have shown that the processing of dynamic emotional signals could be moderated by stable perceiver characteristics, such as empathic personality traits (e.g., Mailhot et al., 2012).

Psychiatric conditions are considered as moderators of dynamic emotional signal decoding. Okruszek reviewed evidence regarding the decoding performance of patients with various psychiatric conditions, such as schizophrenia, in the context of point-light bodily displays. They found that these patients have unique problems, though the magnitude is weaker than impairments in facial or vocal signal processing. Palumbo et al. compared individuals with autism spectrum disorder (ASD) to matched-controls in terms of the ability to evaluate expressions depicted in the last frames of dynamic facial expression videos. The results, together with their previous finding (Palumbo et al., 2015), suggested that ASD impairs the ability to anticipate immediate future emotional state of others' minds. Other studies have reported that individuals with ASD experience other types of impairments in the processing of dynamic facial expressions such as reduced facial mimicry (Rozga et al., 2013).

The modulatory effects of psychiatric conditions and the underlying neural mechanisms in the decoding of dynamic emotional signals are another topic of scientific interest. Sato et al.'s fMRI study investigated brain activity during the observation of dynamic facial expressions in individuals with ASD and typically developing controls. Atypical modulatory influences were found from the amygdala to the neocortical network, including the AON, during the processing of dynamic facial expressions in the ASD group. This corroborates previous findings showing decreased activity and connectivity within the AON during dynamic facial expression processing in individuals with ASD (Sato et al., 2012), which has been proposed to be a core issue associated with ASD (Williams et al., 2001). Other research has reported patterns of brain activity in response to dynamic emotional signals to differ among various psychiatric conditions, including schizophrenia (e.g., Russell et al., 2007).

## Encoding of Dynamic Emotional Signals

Studies have begun to explore the encoding of dynamic facial expressions of emotion, which is generally more difficult to assess than the decoding processes. Scherer et al. analyzed the encoding of emotional facial expressions by actors and found that spatial and temporal patterns of facial action units (AUs; Ekman et al., 2002) are largely consistent with dynamic processes as hypothesized by the component process model (Scherer, 2001). Furthermore, the AU patterns are systematically related to the recognition of emotions in decoders. Hyniewska et al. analyzed the AUs of emotional facial expressions, unobtrusively filmed in a real-life emotional situation, and obtained decoder ratings of emotions and appraisals for these expressions. Associations between specific emotions/appraisals and sets of AUs were found, which suggests that the decoding of emotions/appraisals is achieved via the perception of a set of AUs. Grossard et al. investigated the encoding of emotional facial expressions using different tasks (e.g., imitation of a model) and in different regions using a large sample of children. The results suggested that the encoding of emotional facial expressions is a complex developmental process influenced by several factors (e.g., age).

A few previous studies have investigated the neural mechanisms underlying the encoding of dynamic emotion signals. Heller et al. (2014) simultaneously measured fMRI and facial EMG data during the observation of emotional images and found amygdala activity associated with brow muscle activity in response to negative pictures. In the case of some neural lesions affecting higher level motor control, it is possible to retain capacity for emotional expression in the presence of voluntary facial paresis (e.g., Hopf et al., 1992).

## Other Dynamic Aspects of Emotional Communication

The investigation of dynamic, dyadic interactions remains an understudied and interesting field of research. To demonstrate the dynamic nature of emotional communication, Hareli et al. investigated how an observer's perception of power could be influenced by an emotional exchange between members of a dyad. The results revealed that the perception of power changes depending on the emotional response of one's partner. A previous fMRI study has measured the brain activity of two individuals during face-to-face interactions and observed inter-individual synchronized activity in the lateral occipitotemporal cortex (Koike et al., 2019).

The dynamic perspective can also be applied to the analysis of emotion communication data. Guérin-Dugué et al. jointly recorded ERPs and eye movements during the observation of static emotional facial expressions and applied general linear models to depict the temporal dynamics of neural facial expression processing. Their analyses revealed the emotion-dependent modulation of early components (starting at 20 ms) related to eye fixation in response to facial expressions.

## Conclusions

Together, these findings indicate that a dynamic perspective on emotional communication can provide valuable information. Specifically, the psychological and neural decoding of dynamic facial and bodily signals implies a number of features that differ from those of static displays. Several unique moderators are related to the processing of dynamic emotional messages. Investigation of dynamic facial and bodily expressions are necessary to reveal how emotional messages are encoded. The dynamic perspective can be applied to a broader range of research. Further research should investigate dynamic emotional communication to deepen our understanding of real-life emotional communication.

## Author Contributions

All authors listed have made a substantial, direct and intellectual contribution to the work, and approved it for publication.

### Conflict of Interest

The authors declare that the research was conducted in the absence of any commercial or financial relationships that could be construed as a potential conflict of interest.
